# Emerging Potential Therapeutic Targets of Ferroptosis in Skeletal Diseases

**DOI:** 10.1155/2022/3112388

**Published:** 2022-07-30

**Authors:** Xin Liu, Tianhao Wang, Wei Wang, Xiaolong Liang, Yating Mu, Yaozeng Xu, Jiaxiang Bai, Dechun Geng

**Affiliations:** ^1^Department of Orthopedics, The First Affiliated Hospital of Soochow University, 188 Shizi Street, Suzhou, Jiangsu 215006, China; ^2^School of Chemical Engineering and Technology, Tianjin University, Yaguan Road 135, Tianjin 300350, China; ^3^Orthopedics Institute, Medical College, Soochow University, Suzhou 215000, China; ^4^State and Local Joint Engineering Laboratory for Novel Functional Polymeric Materials, Soochow University, Suzhou, 215123, China

## Abstract

Ferroptosis is a new programmed cell death characterized by the accumulation of lipid peroxidation mediated by iron and inflammation. Since the transcentury realization of ferroptosis as an iron-dependent modality of nonapoptotic cell death in 2012, there has been growing interest in the function of ferroptosis and its relationship to clinical diseases. Recent studies have shown that ferroptosis is associated with multiple diseases, including degenerative diseases, ischemia reperfusion injury, cardiovascular disease, and cancer. Cell death induced by ferroptosis has also been related to several skeletal diseases, such as inflammatory arthritis, osteoporosis, and osteoarthritis. Research on ferroptosis can clarify the pathogenesis of skeletal diseases and provide a novel therapeutic target for its treatment. In this review, we summarize current information about the molecular mechanism of ferroptosis and describe its emerging role and therapeutic potential in skeletal diseases.

## 1. Introduction

Ferroptosis is a new cell death mode characterized by the accumulation of lipid peroxidation mediated by iron. In 2012, Dixon et al. first proposed the definition of ferroptosis, an iron-dependent nonapoptotic mode of cell death characterized by the accumulation of lipid reactive oxygen species (ROS) [1, 2]. Recent studies have shown that ferroptosis is obviously distinct from previous cell death patterns, such as autophagy, necrosis, and necrotic apoptosis, at genetic and characterized levels (Table 1) [1–3]. Unlike the morphological features of necrosis, it does not have membranolytic properties or swelling of the cytomembrane and cytoplasm. Furthermore, in contrast with autophagy, ferroptosis is characterized by rupture of the cell membrane. Specifically, ferroptosis is morphologically shown by reduced mitochondrial cristae and rupture of the mitochondrial outer membrane, which leads to mitochondrial dysfunction [1, 4, 5].

**Figure 1:**
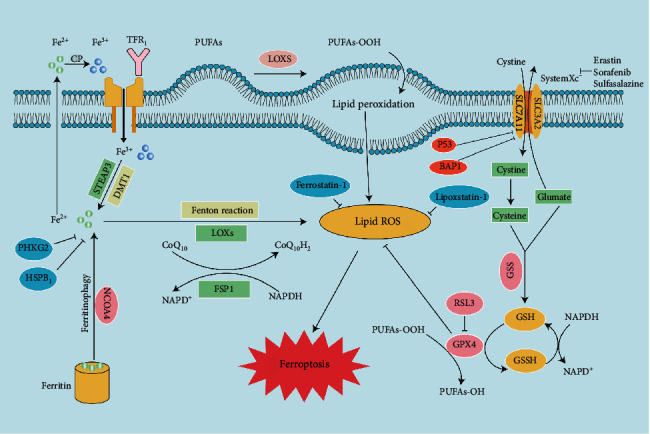
Mechanisms of ferroptosis. Ferroptosis is characterized by iron accumulation, excessive production of ROS, and lipid peroxidation. This illustration shows the process of ferroptosis, summarizing the key molecules and targets regulating iron and lipid peroxidation. TFR1: transferrin receptor 1; PUFA: polyunsaturated fatty acid; LOX: lipoxygenase; STEAP3: six-transmembrane epithelial antigen of prostate 3 metalloreductase; SLC7A11: solute carrier family 7 member 11; DMT1: divalent metal transporter 1; SLC3A2: solute carrier family 3 member 2; BAP1: BRCA1-associated protein 1; ROS: reactive oxygen species; FSP1: ferroptosis suppressor protein 1; FPN1: ferroportin 1; GPX4: glutathione peroxidase 4; GSH; glutathione; GSSG: oxidized glutathione; GSS: glutathione synthetase; PHKG2: phosphorylase kinase G2; HSPB1: heat shock protein beta-1; NCOA4: nuclear receptor coactivator 4; RSL3: Ras-selective lethal 3.

Iron homeostasis and lipid peroxidation are committed steps in the process of ferroptosis (Figure 1) [2, 6]. Superfluous iron induces ferroptosis by producing ROS, while suppressing GPX4 can inhibit ferroptosis through the accumulation of intracellular lipid peroxide [1, 6, 7]. In addition, upon exposure to some compounds used in experiments and clinics, such as erastin, sorafenib, lanperisone, and Ras-selective lethal small molecule 3 (RSL3), ferroptosis was promoted, while ferrostatin-1 (Fer-1), liproxstatin-1 (Lip-1), and zileuton inhibited ferroptosis [8–10]. Current studies have indicated that the relationship between ferroptosis and orthopedic diseases has also attracted extensive attention [1, 6, 11–16]. Ferroptosis has been reported in osteosarcoma cells, promoting their sensitivity to cisplatin with the application of erastin or RSL3, thus attenuating resistance of osteosarcoma to cisplatin *in vitro* [17]. Another report showed that iron overload induced by erythrocyte rupture and the increasing excitatory toxicity of glutamate induced by stress in acute spinal cord injury (SCI) lead to ferroptosis, while desferrioxamine (DFO), an inhibitor dampening ferroptosis, can improve SCI [18]. In view of these findings, this review summarizes recent research progress on ferroptosis to supply references for further understanding of its mechanism and describe its emerging role in skeletal diseases.

## 2. Origin and Development

The concept of programmed cell death first emerged in the 1960s, before ferroptosis was defined [19, 20]. Previous studies on cell death indicated that GPX4, the fourth member of the selenium containing GPX family, but not GPX1, reduces lipid hydroperoxides in membranes, and cloning GPX4 reveals its distinct nature such as the scavenging capacity of membrane lipid hydrogen peroxide products compared to other GPXs, thus suppressing cell death [21, 22]. It was thought that this phenomenon was caused by the inhibition of apoptosis or autophagy. Meanwhile, the presence of GPX4 has been further observed in animals and humans [23, 24]. This further confirms the important role of GPX4 in cell death patterns. Before long, Dolma et al. found that erastin had selective lethality in Ras-expressing cancer cells, and the cell death pattern was different from what we previously knew until the 2000s [25]. In addition, RSL3 and RSL5, which can directly bind to GPX4 protein to inactivate it and induce the production of lipid ROS, selectively killed Ras-transformed (BJeLR) BJ fibroblasts in a new way different from apoptosis [1]. It is interesting that the mode of cell death induced by this composition is nonapoptotic, as cell death is induced by erastin and RSL3 in the absence of apoptotic markers [4, 5, 25]. Ushered in a major breakthrough in 2012, the new terminology ferroptosis was coined from nutrient consumption-induced cancer cell death and oxidation, the death of neurons yielding to the glutamate excitotoxin and inhibiting the amino acid antiporter solute carrier family 7 member 11 (SLC7A11/x CT/system Xc^−^) [26–29]. With the discovery of this concept, subsequent studies confirmed the phenomenon that GPX4 inhibited iron-mediated death by inhibiting ROS production rather than autophagy or apoptosis, as previously thought. Recent studies have identified lipophilic antioxidants as powerful inhibitors of erastin-induced cell death, suggesting that ROS are involved in this cell death process (Figure 1) [5, 30]. Moreover, iron chelating agents were identified as suppressors of cell death induction after RSL3 treatment, suggesting the requirement for cellular iron [4].

**Figure 2:**
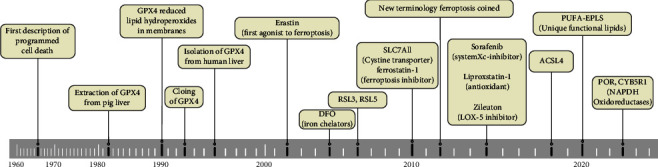
The timeline of ferroptosis.

In 2014, Skouta et al. found that Fer-1, a chemical compound inhibiting the peroxidation induced by iron and trace lipid hydroperoxide in liposomes, suppressed cell death in some disease “models,” such as Huntington's disease (HD), periventricular white matter (PVL), and renal insufficiency. This discovery provides the basis for the use of ferrostatin in models of diseases, and it is the first to stress the importance of ferroptosis beyond the cellular level [31]. In 2015, new results revealed that the retinoblastoma- (Rb-) negative status of hepatocellular carcinoma cells promotes iron oxidation disease (a type of oxidative necrosis) after exposure to sorafenib. These findings highlight the role of retinoblastoma in the response of HCC cells to sorafenib and the regulation of iron disease [32]. Subsequent studies showed that the iron chelating agent deferoxamine (DFO) significantly inhibited RSL3/BV6-induced cell death, but it did not protect erastin/BV6 cells from death, suggesting that RSL3/BV6- and not erastin/BV6-mediated cell death depended on iron in 2017 [33]. In addition, activation of the Nrf2-ARE pathway contributed to HNV cell resistance to GPX4 inhibition, and inhibition of the Nrf2-ARE pathway reversed resistance to iron transformation in HNC cells in 2018 [34]. In 2020, glutamine deprivation increased vorinostat-mediated cell death and ROS accumulation, and genetic elimination of xCT improved the efficacy of vorinostat by inducing ferroptosis (Figure 2) [35]. In summary, the discovery and development of a new death mode named ferroptosis may provide a research approach to learn and treat multidisciplinary diseases.

## 3. The Relationship between Bone Metabolism and Iron

Iron is an important trace element in nature. In recent years, people have gradually realized that iron deficiency and iron overload are important inducers of the occurrence and development of ferroptosis. For the sake of a deeper understanding of bone metabolism and ferroptosis, researchers have begun to explore the relationship between iron and bone metabolism. Medeiros et al. found that bone volume fraction (BV/TV) and bone trabecular thickness decreased with the increment of bone trabecular separation analyzed by micro-CT in a female rat model fed an iron-deficient diet [36]. With the exception of the effects of iron deficiency on bone structure, markers of bone transformation were also affected, which may provide ideas into the detailed mechanism of how iron deficiency affects bone. In contrast, the expression levels of parathyroid hormone (PTH) and tartrate-resistant acid phosphatase 5B (TRAP) were upregulated in iron-deficient rats, suggesting that severe iron deficiency leads to increased bone resorption as bone formation decreases. In addition, previous studies have shown that the mineralization function of osteoblasts is damaged through a lack of iron [37]. A population study also reported that iron deficiency anemia (IDA) played a significant role in some bone health indicators. In a population-based study from Taiwan, the risk ratio of IDA to osteoporosis was 1.74 higher than that of individuals with normal iron status [38]. Contrary to common sense, the more iron there is, the better. High levels of iron in menopausal female and some genetic diseases are correlated with a decrease in bone quality and activity. These processes eventually lead to loss of bone mass, increased risk of bone fracture, and formation of osteoporotic bone phenotypes, as stated elsewhere [39]. Although the effects of menopause were excluded, Kim et al. also found that an increase in ferritin resulted in bone loss [40]. Subsequent studies have shown that there is a relationship between iron overload and a high incidence rate of osteoporosis in hereditary hemochromatosis (HH) [41, 42]. As described above, bone metabolism is disordered in an environment of iron deficiency, which leads to the occurrence of orthopedic diseases.

## 4. Inducers

To date, there are many inducers of ferroptosis (Table 2). We briefly describe the following compounds.

### 4.1. Erastin

Cell death induced by erastin is efficiently suppressed by antioxidants such as *α*-tocopherol, *β*-carotene, butylated hydroxytoluene, and iron chelators, indicating that ferroptosis induced by erastin requires ROS- and iron-dependent signaling [1, 43]. Upregulation of RAF/MEK/ERK signaling can be significant for ferroptosis induced by erastin in tumor cells carrying oncogenic Ras [43]. *In vivo*, the adaptation and water solubility of piperazine erastin are better than those of erastin in suppressing cancer growth [44].

The mitochondrial voltage-dependent anion channel (VDAC) is one of the direct molecular targets of erastin, which can be directly attached to VDAC2/3 in BJeLR cells [43]. Reducing the expression of VDAC2 and VDAC3, instead of VDAC1, brings about erastin resistance [43]. In addition, erastin is able to decrease glutathione (GSH) levels by directly suppressing system Xc^−^ activity to affect the cystine/glutamate antiporter, activating the stress response in the ER in bone marrow-derived mesenchymal stem cells [1, 45, 46]. This response will accelerate ROS accumulation in ferroptosis.

### 4.2. RSL3 and RSL5

In tumor cells, ROS, iron, and MEK are essential in ferroptosis induced by RSL3 and RSL5 [4]. VDAC2/3 is necessary for RSL5 to realize ferroptosis but not for RSL3 [4]. RSL3 can directly inhibit GPX4 [7]. After integrating into GPX4, RSL3 suppresses GPX4 to promote lipid peroxidation to increase ROS production [7]. Therefore, recent studies have shown that at least two types of RSLs exist. Type I RSLs, such as erastin and RSL5, can trigger ferroptosis by targeting upstream regulators. Type II RSLs suppress downstream regulators such as GPX4 to induce ferroptosis. RSL5-induced ferroptosis can be inhibited by a protein synthesis inhibitor but not RSL3-induced ferroptosis, indicating that type I RSL-induced ferroptosis requires protein synthesis.

### 4.3. Lanperisone

Lanperisone, an improved version of tolperisone, has been developed as a skeletal muscular relaxant [45]. Lanperisone can selectively kill K-Ras-mutant mouse embryonic fibroblasts through the induction of ROS mediated through iron and Ras/RAF/MEK/ERK signaling. In addition, lanperisone may inhibit the function of system Xc^−^ or other targets in ferroptotic pathways [1]. LP appears to be similar to erastin in terms of potential mechanisms. By binding to mitochondrial voltage-dependent anion channels (VDACs), erastin alters VDAC gating, leading to mitochondrial dysfunction and ROS production and ultimately inducing ferroptosis. Lanperisone also suppresses tumor growth in a K-Ras-driven mouse model of lung cancer *in vivo* [47]. The specific mechanism of ROS generation induced by lanperisone is not clear, but a study suggests that the interference of voltage-gated ion channels is important [45].

### 4.4. Sorafenib

Sorafenib can induce ferroptosis in some cancer cells, such as colorectal cancer cells, hepatoma cells, and osteosarcoma cell [32, 48, 49]. Ferroptosis induced by sorafenib occurs free from the carcinogenic state [50]. However, the expression of Nrf2 and Rb can suppress ferroptosis induced by sorafenib in HCC [32, 51]. The function of sorafenib in ferroptosis may be associated with the inhibition of system Xc^−^ instead of GPX4 expression. This process is linked to the upregulation of ER stress [52]. Further research on sorafenib analogs shows that sorafenib suppresses system Xc^−^ via a nonkinase target [52].

## 5. Inhibitors

In addition to the above, we also summarized the inhibitors of ferroptosis classified by some targets (Table 3).

### 5.1. Ferrostatin

Initial ferrostatin is known as ferrostatin-1 (Fer-1) and acts as an inhibitor of ferroptosis induced by RSL3 and erastin in fibrosarcoma HT-1080 [1]. The activity of Fer-1 is determined by the primary aromatic amine, which particularly suppresses lipid oxidation and decreases the accumulation of ROS [1]. In addition, Fer-1 can inhibit osteoblast ferroptosis by regulating the Nrf2-ARE signaling pathway, thereby alleviating nanoparticle-induced peri-implant osteolysis [53]. In comparison with Fer-1, new-generation ferrostatins (SRS 11–92 and SRS 16–86) have the advantages of improving the stability of metabolism and tremendously preventing diseases such as acute kidney injury and ischemia-reperfusion injury [54, 55].

### 5.2. Liproxstatin-1

Liproxstatin-1 (Lip-1), a potent spiroquinoxalinamine derivative, is known to inhibit the accumulation of ROS from lipid oxidation and cell death in GPX4 knockout cells [43]. Lip-1 may function as a lipophilic antioxidant, although the mechanism of action of this inhibitor has yet to be reported. Previous studies have proven the close relationship between Lip-1 and ferroptosis. *In vitro*, Lip-1 inhibits ferroptosis induced by ferroptosis inducers such as erastin, RSL3, and buthionine sulfoximine. Recent studies have also found that Lip-1 suppresses ferroptosis in osteosarcoma cells induced by bavachin through the stat3/p53/slc7a11 axis [56]. Intraperitoneal injection of Lip-1 in GPX4 knockout mice prolongs animal life in response to renal injury [43]. Lip-1 can also prevent other organ ischemia reperfusion injuries in mice, such as hepatic and cerebral injury by evaluating proferroptotic changes after ischemia and the levels of protein and lipid peroxidation [57].

### 5.3. Prominin-2

Ferroptosis causes clinically extensive necrosis during heart attack and acute kidney injury. Belavgeni et al. described the terpenoid membrane glycoprotein prominin-2 as a novel endogenous ferroptosis inhibitor [58]. Subsequent studies found that iron-promoting stimulants (including inhibition of GPX4 and its withdrawal from the extracellular matrix) induce the expression of prominin2, a pentapeptide involved in the dynamic regulation of the vitamin. Prominin2 promotes iron-resistant death of breast epithelial and breast cancer cells [59]. Mechanistically, prominin2 promotes the formation of ferritin-containing polyvesicles (MVBs) and exosomes that transport iron out of the cell, thereby inhibiting ferroptosis. These findings suggest that resistance to iron death can be driven by the prominin2-MVB-exosome-ferritin pathway and has broad implications for iron homeostasis, intracellular transport, and cancer [59, 60].

## 6. Important Mechanisms Associated with Ferroptosis

### 6.1. Inducing Ferroptosis by Suppressing System Xc^−^

System Xc^−^ is a plasma membrane cystine/glutamate antiporter made up of a twelve-pass transmembrane transporter protein linked to the transmembrane regulatory protein SLC3A2 through a disulfide bridge that is often present in phospholipid bilayers. It is also an integral part of the cell antioxidant system. Cystine and glutamate are intercellularly and extracellularly exchanged by system Xc^−^ at equal proportions [1]. Cysteine is degraded into cysteine in cells and is closely related to the production of glutathione (GSH). GSH suppressed ROS in the presence of glutathione peroxidases (GPXs). Suppressing the viability of system Xc^−^ influences the production of GSH by decreasing the absorption of cystine, which results in downregulating GPX activity and greatly weakening cell antioxidant capacity. Due to the influence described above, the eventual occurrence of oxidative damage and ferroptosis is inevitable. In addition, by downregulating the expression of SLC7A11, p53 can also inhibit cystine uptake by system Xc^−^, thus reducing the activity of GPX4, leading to decreased cell antioxidant capacity and ferroptosis [61, 62].

### 6.2. Inducing Ferroptosis by Suppressing GPX4 through the MVA Pathway

In the case of the GPX family, recent studies have shown that GPX4 plays a considerable role in the occurrence of ferroptosis mainly by suppressing the formation of lipid peroxides. GPX4 is a unique selenium-utilizing form of glutathione peroxidase that can specifically protect lipids in cell membranes from oxidative damage. GPX4 acts as a phospholipid hydroperoxidase and plays a role in the conversion of glutathione to oxidized glutathione (GSSG) and decreases phospholipid hydroperoxide production (AA/ADA-PE-OOH) to the corresponding phospholipid alcohol (PLOH) [63]. The accumulation of lipid peroxides induced by the downregulation of GPX4 activity can result in the emergence of ferroptosis. In contrast, cells overexpressing GPX4 suppress ferroptosis by decreasing the production of lipid peroxides [43]. RSL3, which induces ferroptosis, has a direct function on GPX4 and suppresses its activity to reduce the generation of the antioxidant capacity of cells and accumulate ROS, thus resulting in the occurrence of ferroptosis [7]. Selenocysteine is one of the essential amino acids in the activation of GPX4, and its tRNA, which plays a crucial role in efficient translational decoding of UGA and synthesis of selenoproteins through isopentenylation, links selenocysteine to GPX4 [64]. The mevalonate (MVA) pathway is a metabolic pathway for the synthesis of isoprene pyrophosphate and dimethyl allyl pyrophosphate from acetyl coenzyme A. In addition, when GPX4 is inactivated, it will inhibit the conversion of lipid peroxide to lipid alcohol. Lipid peroxide (LPO) can be used as an important indicator of ferroptosis. For example, nanoplatform could inhibit the expression of HMGCR to downregulate the mevalonate (MVA) pathway and glutathione peroxidase 4 (GPX4), thereby producing more LPO to induce cancer cell ferroptosis. Apart from these, IPP and CoQ_10_ play vital roles in the mevalonate (MVA) pathway [64–66]. Consequently, suppressing the MVA pathway can reduce the synthesis of selenocysteine tRNA, thus influencing GPX4 expression and leading to ferroptosis.

### 6.3. Inducing Ferroptosis by Injuring Mitochondrial VDACs

VDAC is involved in energy metabolism mainly by affecting the transport of ATP/ADP in and out of mitochondria. Meanwhile, VDACs, as transmembrane channels, can transport ions and metabolites and play a key role in regulating ferroptosis [67]. Tarangelo et al. found that erastin exerts effects on VDACs, leading to mitochondrial structural injury and dysfunction and bringing about a large amount of released reactive oxygen species (ROS), ultimately leading to ferroptosis [68]. By blocking the oligomerization of VDAC1 but not VDAC2 or VDAC3, they found that liproxstatin-1 short-circuited the ferroptosis pathway [69].

### 6.4. Ferroptosis Mediated by p53

p53 is a pivotal tumor suppressor gene, which regulates cell growth and senility by promoting apoptosis and repairing DNA under stressful conditions and plays a vitally important role in the occurrence and development of tumors [70]. Researchers have accidentally found that p53 is also closely related to ferroptosis. A study has shown that acetylation-deficient p53 mutants can boost ferroptosis. Jiang et al. found that the activity of p53-silenced H1299 cells was not different when induced by ROS [61]. However, almost all of the cells died induced by reactive oxygen species after activating p53. The cell death rate decreased significantly when cells were treated with Fer-1, a ferroptosis inhibitor. Recent studies have reported that p53 can suppress cystine uptake by system Xc^−^ through downregulation of SLC7A11 expression, thus regulating GPX4 expression and reducing antioxidant capacity and ultimately ferroptosis [61]. In addition, the p53-SAT1-ALOX15 pathway is also associated with the process [67]. In addition, the expression of p53 can also have an opposite effect on ferroptosis. Tarangelo et al. found that p53 suppressed the competence of system Xc^−^ but also decreased the sensitivity of many cells, such as human HT-1080 fibroblasts, to ferroptosis [68]. He et al. found that these cells, called human HT-1080 fibroblasts, were not sensitive to ferroptosis induced by erastin-2 when treated with the p53 inducer nutlin3. Subsequent research demonstrated that nutlin-3 increases the expression of wild-type p53 in wild-type U-2OS, Caki-1, and A549 cells, which could lead to ferroptosis [71]. In addition, inhibition of ferroptosis sensitivity requires the help of CDKN1A (encoding p21), which regulates glutathione synthesis and metabolism. Previous outcomes clearly state that the p53-p21 axis can negatively regulate ferroptosis in cancer cells [68]. Apart from what was mentioned before, Xie et al. found that p53 played an important role in suppressing ferroptosis in colorectal cancer [72]. Consequently, p53 regulation of ferroptosis may be bidirectional and the detailed mechanism needs to be further studied.

### 6.5. Relationship between Iron Metabolism and Ferroptosis

Iron is a necessary trace element for humans. Abnormal iron metabolism in the body can affect the occurrence and development of normal physiological processes. In the blood, iron mainly exists in the form of Fe^2+^, which originates from erythrocyte degradation or intestinal absorption [73]. Six transmembrane epithelial antigen of the prostate 3 (STEAP3) can resolve Fe^3+^ into Fe^2+^, and unstable iron pool (LIP) and ferritin will finally be storage places for Fe^2+^ with mediation of zinc-iron regulatory protein family 8/14 (ZIP8/14) or divalent metal transporter 1 (DMT1). Superfluous Fe^2+^ is oxidized to Fe^3+^ by ferroportin (FPN) [74]. The recyclability of internal iron, as mentioned above, strictly regulates iron homeostasis in cells. Silencing TFRC (the gene encoding TFR1) can suppress ferroptosis induced by erastin [11], but heme oxygenase-1 (HO-1) can expedite ferroptosis induced by erastin by replenishing iron [75]. Recent studies have found that ferroptosis is related to heat shock protein beta-1 (HSPB1). This protein can suppress TFR1 expression to decrease intracellular iron content, and the overexpression of HSPB1 can effectively inhibit ferroptosis [76]. Iron response element binding protein 2 (IREB2) plays a key role in iron metabolism, and inhibiting it can significantly inhibit ferroptosis by increasing the expression of ferritin heavy chain 1 (FTH1), which is an important component of ferritin [77].

### 6.6. Regulating Ferroptosis through the Lipid Metabolism Pathway

The accumulation of ROS is one of the important characteristics of ferroptosis. Lipid metabolism and ferroptosis are closely related. The lipid peroxidation of polyunsaturated fatty acids (PUFAs) is susceptible and closely related to the occurrence of ferroptosis [78]. The process of esterification and oxidation is necessary to transmit signals to cause ferroptosis. Recent research shows that phosphatidylethanolamine (PE), containing arachidonic acid (AA) or its derivative adrenaline, plays a pivotal role in ferroptosis [79]. Lysophosphatidylcholine acyltransferase 3 (LPCAT3) and acyl-CoA synthetase long-chain family member 4 (ACSL4) are related to the synthesis and reconstruction of PE, activating PUFAs and influencing the transmembrane function of PUFAs [80]. Therefore, downregulating the expression of the above two products can reduce the accumulation of intracellular lipid peroxide substrates and inhibit ferroptosis. Ultimately, with the catalysis of lipoxygenase (LOX), PUFA-PE can play a further oxidative role and eventually induce ferroptosis.

## 7. Ferroptosis in Bone-Related Diseases

### 7.1. Osteoporosis

Osteoporosis is a systemic bone disease that is prone to fracture due to the decrease in bone density and quality, destruction of bone microstructure, and increase in bone fragility [81]. In addition, pain caused by osteoporosis can reduce the quality of life of patients; spinal deformation and fracture can be disabled, limiting patients' activities and increasing the incidence of pulmonary infection and bedsores, not only increasing the quality of life and mortality of patients but also imposing a heavy economic burden on individuals, families, and society [82]. Current treatments for osteoporosis are limited, and calcium is only supplemented when appropriate, but calcium alone cannot be used as an osteoporosis treatment, only as a basic adjunct, and an increasing number of people are experiencing the problem, so addressing this aspect is imminent [83]. In addition, previous studies on autophagy, apoptosis and osteoporosis have some shortcomings [84, 85]. The discovery of ferroptosis may provide a new direction for the treatment of osteoporosis.

**Figure 3:**
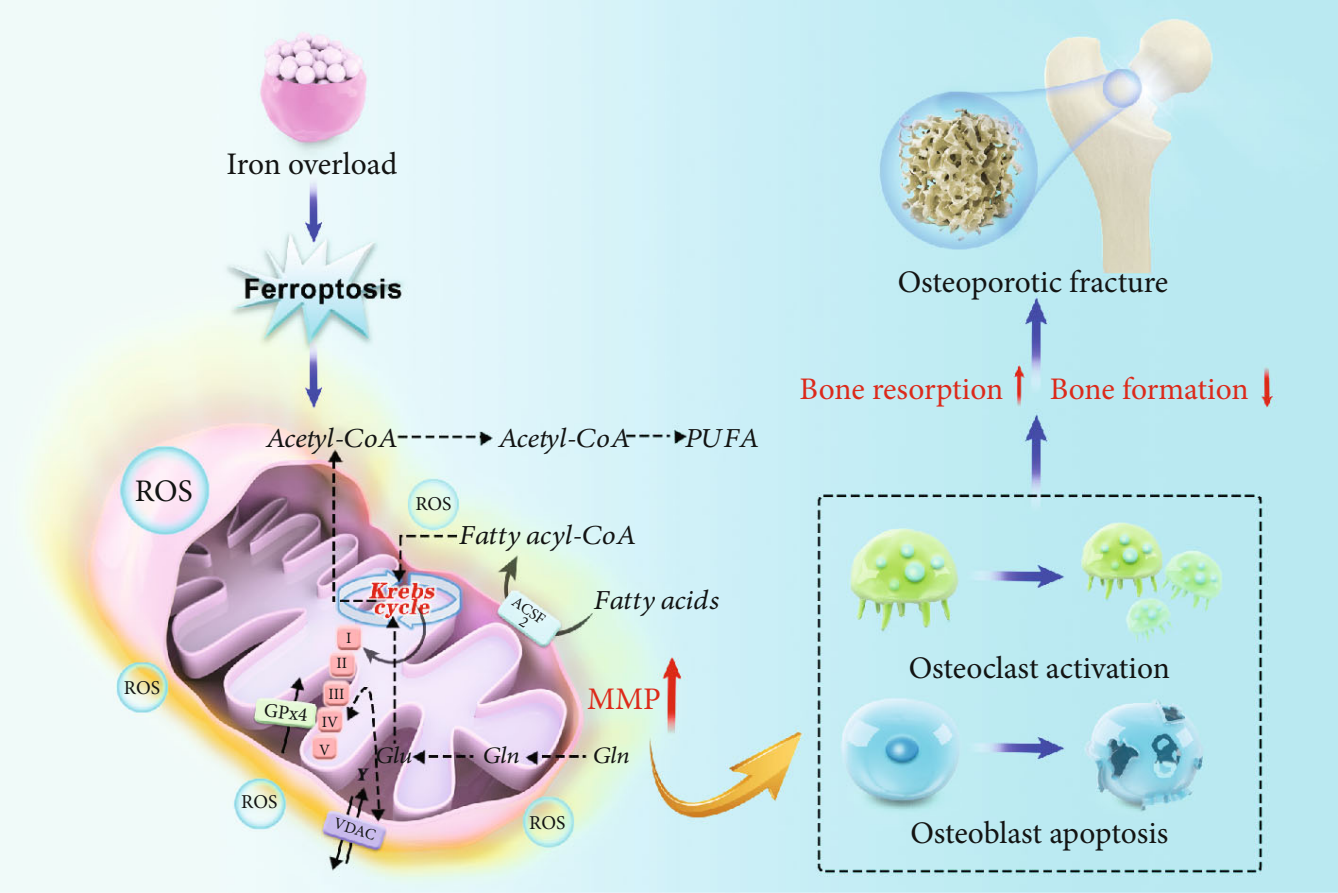
The mechanism of ferroptosis induced by iron overload in osteoporosis. Ferroptosis induced by iron overload leads to an increase in mitochondrial membrane potential and the accumulation of lipid peroxide by affecting glutathione and fatty acid cycle. It further promotes the activation of osteoclasts and the apoptosis of osteoblasts, leading to the increase of bone resorption and the decrease in bone formation, resulting in osteoporosis and finally osteoporotic fracture.

Along with the further comprehension of ferroptosis, ferroptosis is recognized as a new factor for osteoporosis. Tian et al. observed that the death of osteoblasts *in vitro* induced by ferroptosis is involved in the mitochondrial apoptotic pathway through the analysis of intracellular labile iron levels by flow cytometry and fluorescence microscopy and mitochondrial membrane potential (MMP) [86]. Iron overload could induce apoptosis in osteoblasts and osteoporosis *in vivo*. Mitochondrial apoptosis and ROS-mediated necroptosis are closely related to ferroptosis, leading to the death of osteoblasts and thus causing bone rarefaction [87, 88]. In addition, some experts found that the characteristics of ferroptosis such as the function of osteoblasts and osteoclasts in osteoporotic mice are reflected in bone mineral density, trabecular number, and trabecular bone mass measured by micro-CT in the femur [89]. The above studies suggest that ferroptosis has a great influence on osteoporosis (Figure 3).

Recently, Ni et al. observed that ferroptosis affected osteoclasts in the process of differentiation induced by RANKL. The overexpression of TFR1 and the amount of significantly decreased ferritin induced by downregulating aconitase activity can lead to ferroptosis during RANKL stimulation without oxygen deficiency. However, these phenomena regarding changes in intracellular iron homeostasis and the activation of ferritinophagy could not be observed under hypoxia. In addition, they also found that HIF-1*α* impaired autophagic flux under hypoxia *in vitro*. 2ME2, a HIF-1*α*-specific inhibitor, prevents OVX-induced osteoporosis in rats *in vivo*, probably due to the increase in ferroptosis markers such as PTGS2 and MDA [90]. In addition, other scholars found interesting phenomena about the occurrence of ferroptosis in skeletal muscle related to changes in iron metabolism and lipid peroxidation and different expression of TFR1 by comparing different age groups of mice. With intramuscular injection of lentivirus expressing TFR1, skeletal muscle regeneration is enhanced and suppresses ferroptosis in different age groups of mice, thus preventing osteoporosis [91]. Liu et al. further clarified the relationship between ferroptosis and osteoporosis through animal models of osteoporosis *in vivo* and cell models *in vitro* [92]. From the above research content, we have a better understanding of ferroptosis orchestrated with osteoporosis than before and can treat and prevent osteoporosis according to some targets. However, due to some technical limitations, many detailed mechanisms in ferroptosis involved in osteoporosis remain unclear.

### 7.2. Acute Spinal Cord Injury

SCI is usually caused by fractures and/or dislocations of the spine as a result of direct or indirect violence. It leads to severe dysfunction of the lower limb and thus causes serious physical and psychological damage to patients themselves and causes a huge economic burden on society. SCI has high mortality and disability rates [93]. Due to previous research on SCI, the prevention, treatment, and rehabilitation of SCI have become major topics in today's medical community.

Previous studies have mostly studied the mechanisms of acute spinal cord injury from the aspects of apoptosis and autophagy. For example, the AMPK/mTOR signaling pathway is activated after spinal cord injury. This inactivates the intracellular AMPK-activated mTOR, which catalyzes the phosphorylation of ULK1 to promote autophagy. After spinal cord injury, the mitochondria of neurons produce excessive reactive oxygen species through the processes of protein decomposition, lipid peroxidation, and DNA damage, which leads to the aggravation of spinal cord injury and apoptosis [94, 95]. In addition, potential roles of phenolic compounds as key phytochemicals have also been revealed in preclinical and clinical studies in regulating upstream dysregulated oxidative stress/inflammatory signaling mediators and extrinsic mechanisms of axon regeneration after SCI [96, 97]. Meanwhile, polyphenols were also identified as a potent inhibitor of ferroptosis, which was confirmed in *in vitro* and *in vivo* studies in different disease models [98–101]. However, whether polyphenols can improve acute SCI by interfering with ferroptosis and whether they interact with apoptosis and autophagy still need further study. Fortunately, recent studies have shown that variances in mitochondrial function and structure in ferroptosis can be observed by transmission electron microscopy, and ferroptosis markers in SCI rats exhibit several changes in spinal cord tissue, which are different from autophagy and apoptosis. As mentioned above, ferroptosis plays an important role in SCI [18]. After SCI, spinal cord hemorrhage, degeneration, red blood cell rupture, and hemolysis occurred in the injured spinal cord and thus caused iron overload. Stress also activated ROS accumulation and lipid peroxidation [102].

Some scholars have found that ferroptosis can lead to serious consequences of secondary injury after spinal cord injury and that DFO can suppress ferroptosis to promote functional recovery in SCI rats [18]. Galluzzi et al. experimented on spinal nerve cells with ferrous ions and found that with the increase in iron in cells, the degree and metabolites of lipid peroxidation related to neuronal inactivation also increased [103]. Zhang *et al.* observed that a ferroptosis inhibitor called SRS16-86 can reduce ferroptosis markers and upregulate the levels of GPX4, xCT, and GSH in SCI rats, thus preventing more complications after SCI [104]. In addition, the morphology of mitochondria was similar to normal, and more mitochondrial cristae appeared after SRS16-86 intervention. Subsequent studies have shown that the extracellular regulated protein kinase (ERK) pathway has a certain connection with ferroptosis, and downregulating the RAS/RAF/ERK pathway by the ferroptosis inhibitor U0126 could inhibit neuroinflammation and protect neurons, thus recovering from SCI and reducing local redox damage [105]. As will be readily seen from what we mentioned above, acute SCI is closely related to ferroptosis. However, the current research is still insufficient, and the detailed mechanism of SCI related to ferroptosis is unclear.

### 7.3. Osteosarcoma

Osteosarcoma is one of the most common bone malignancies. The typical osteosarcoma originates from the bone; another completely different type is osteosarcoma juxtaposed with the bone cortex, which originates from the periosteum and adjacent connective tissue [106]. The mortality and disability rate of osteosarcoma is high in children and adolescents. Some patients suffer from both physical and psychological damage. Although early diagnosis and timely medication or surgery have greatly improved patient quality of life, subsequent physical and mental rehabilitation treatment is limited, and its curative effect is not satisfactory.

**Figure 4:**
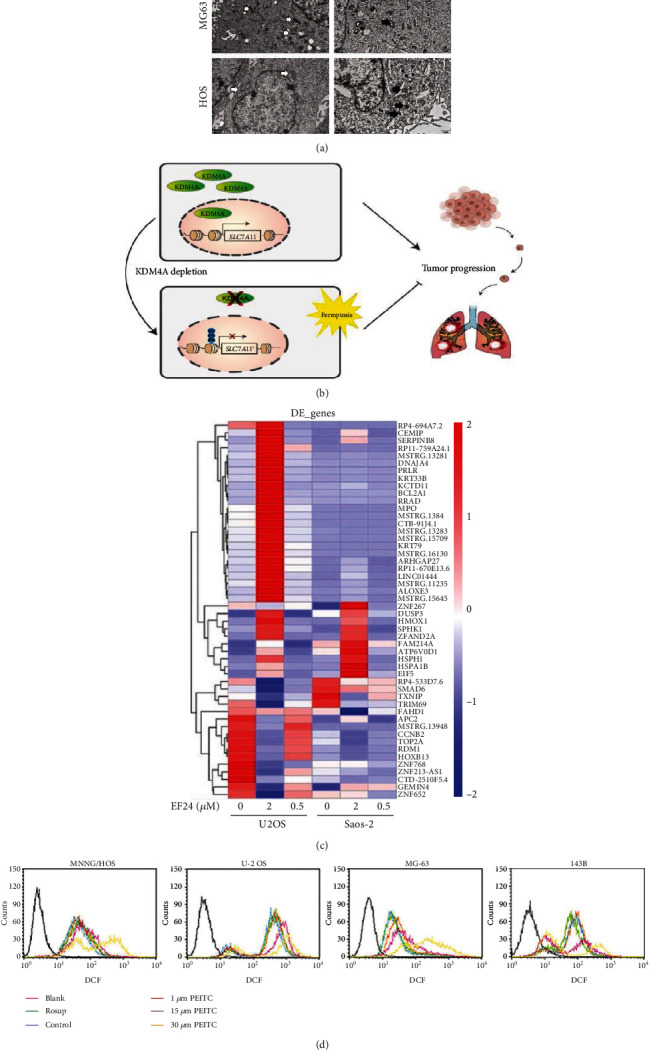
(a) The ultrastructure of MG63 and HOS cells (reproduced from ref. [56] with permission from 2021 Hindawi Publishing Corporation); (b) KDM4A regulation of ferroptosis and tumor progression in OS (reproduced from ref. [107] with permission from 2021 Elsevier B.V.); (c) RNA sequencing analysis of gene transcriptional profiles changings of U2os cells and Saos2 cells after treating with indicated dose of EF24 (reproduced from ref. [108] with permission from 2021 Elsevier B.V.); (d) ROS levels in MNNG/HOS, U-2 OS, MG-63, and 143B cells treated with PEITC for 24 h (reproduced from ref. [110] with permission from 2020 Hindawi Publishing Corporation).

Gratifyingly, bavachin, a bioactive compound extracted from the fruit of Psoralea corylifolia, induces ferroptosis through the STAT3/p53/SLC7A11 axis in osteosarcoma cells, such as MG63 and HOS cells, thus inhibiting the further development of osteosarcoma (Figure 4(a)) [56]. Meanwhile, Chen et al. found that ferroptosis can lead to lipid peroxidation and dysfunction after osteosarcoma and that Fer-1 could suppress ferroptosis to promote functional recovery in osteosarcoma mice (Figure 4(b)) [107]. Recent studies have shown that osteosarcoma cells, such as U2os and Saos-2 cells, have a high level of ROS and more lipid peroxidation metabolites than normal cells. Coincidently, they observed ferroptosis marker changes in osteosarcoma cells. Lin *et al.* confirmed that a ferroptosis inhibitor named ferrostatin-1 could reduce ferroptosis-related genes such as HMOX1 and upregulate GPX4 expression in osteosarcoma cells after intervention with EF24 (a synthetic analog of curcumin), thus promoting the recovery of cell function and morphology (Figure 4(c)) [108]. In addition, subsequent studies have shown that NF-*κ*B signaling and the mitogen-activated protein kinase (MAPK) pathway have a certain connection with ferroptosis, and downregulating the MAPK pathway by the ferroptosis inhibitor Fer-1 could promote osteosarcoma cell death, thus recovering from osteosarcoma and reducing ROS production (Figure 4(d)) [17, 108–110]. Lv et al. also found that *β*-phenethyl isothiocyanate, a valid medicine against cancers such as lung cancer and breast cancer, could lead to human osteosarcoma cell death by interfering with iron metabolism through upregulating the MAPK signaling pathway [111–113]. As time goes by, people may pay much more attention to osteosarcoma in children and adolescents, and we also have a profound understanding of this disease. However, as far as we know, the role of ferroptosis in the regulation of osteosarcoma is unclear. Further research is needed to elucidate the detailed mechanism of osteosarcoma correlated with ferroptosis.

### 7.4. Osteoarthritis

Osteoarthritis is a degenerative disease that involves the degeneration and injury of articular cartilage and reactive hyperplasia of articular edge and subchondral bone caused by many factors, such as aging, trauma, congenital joint abnormalities, and joint deformities [114]. According to statistics, approximately 300 million people suffer from osteoarthritis worldwide [115]. While surgery and medication have greatly improved the motor functions of patients, subsequent rehabilitation treatment requires further perfection [116].

**Figure 5:**
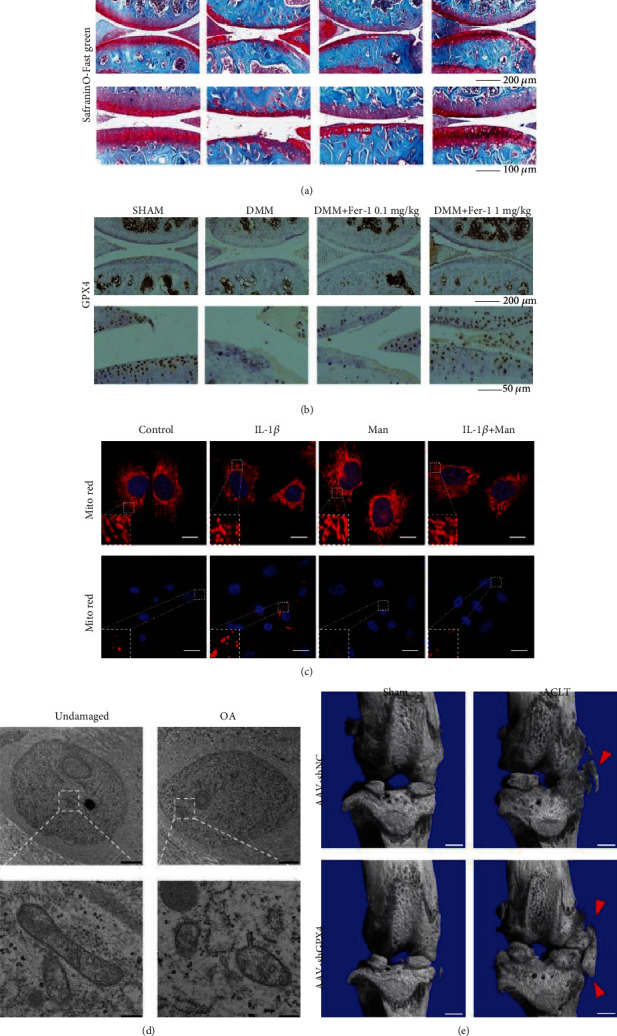
(a) Cartilage degradation was assessed by safranin O/fast green about collagen II and GPX4 expression in an OA model (reproduced from ref. [119] with permission from 2020 Elsevier B.V.); (b) immunohistochemistry staining of GPX4 (reproduced from ref. [119] with permission from 2020 Elsevier B.V.); (c) chondrocytes 24 h postindicated treatments by MitoTracker Red staining (reproduced from ref. [121] with permission from 2021 Ovid Technologies, Inc.); (d) mitochondrial membrane rupture in OA cartilage tissues by a transmission electron microscope (reproduced from ref. [122] with permission from 2022 Elsevier B.V.); (e) three-dimensional models of mouse knee joints. Red arrow shows osteophyte formation (reproduced from ref. [122] with permission from 2022 Elsevier B.V.).

Recent studies have shown that OA, which is closely related to inflammation, is a complex process associated with ferroptosis in terms of iron homeostasis [117, 118]. Yao et al. found that erastin, a specific ferroptosis inducer, downregulated type II collagen (collagen II) expression orchestrated with OA in chondrocytes, while ferrostain-1 could ameliorate this phenomenon by eliminating lipid ROS (Figures 5(a) and 5(b)) [119]. Ferrostatin-1 attenuated OA progression, as detected by immunohistochemistry and the OARSI score, by suppressing ferroptosis and upregulating GPX4 expression in the OA mouse model. In addition, the Nrf2 antioxidant system and ferroptosis regulate each other under inflammatory and iron overload conditions, although the detailed mechanism is still unclear [120]. A subsequent study also found that D-mannose, a compound involved in immune regulation, exerted a chondroprotective effect by attenuating the sensitivity of chondrocytes to ferroptosis and alleviating OA progression (Figure 5(c)) [121]. Through further research, GPx4 was shown to play an important role in the relationship between osteoarthritis and ferroptosis. GPx4 regulates ferroptosis or oxidative stress and ECM degradation through the MAPK/NF-*κ*B signaling pathway to alleviate the progression of osteoarthritis (Figures 5(d) and 5(e)) [122]. Bin et al. also found that inflammation induced by suppressing miR-10a-5p regulated by IL-6 can promote ferroptosis in cartilage cells through cellular oxidative stress and iron homeostasis imbalance [105]. In addition, subsequent results suggest that IL-6 in IVD exacerbates its degeneration by inducing cartilage cell ferroptosis, thus causing lumbar instability, fracture, and intervertebral disc degeneration [123]. They may make the IL-6/miR-10a-5p/IL-6R axis a potential therapeutic target for IDD intervention in the future. To date, we realize that ferroptosis plays an important role in osteoarthritis, but the detailed mechanisms require further study.

### 7.5. Rheumatoid Arthritis

Rheumatoid arthritis (RA), a chronic systemic disease with a sophisticated etiology, is considered a common disease that affects 0.5–1% of the global population [124]. RA is a chronic autoimmune disease that is characterized by multijoint, symmetrical, and invasive joint inflammation and is often accompanied by the involvement of extraarticular organs and positive serum rheumatoid factor, which can lead to joint deformity and loss of function [125, 126]. However, current medical strategies only alleviate symptoms and delay the process instead of healing it completely, and later rehabilitation exercise is not very satisfactory [127, 128].

**Figure 6:**
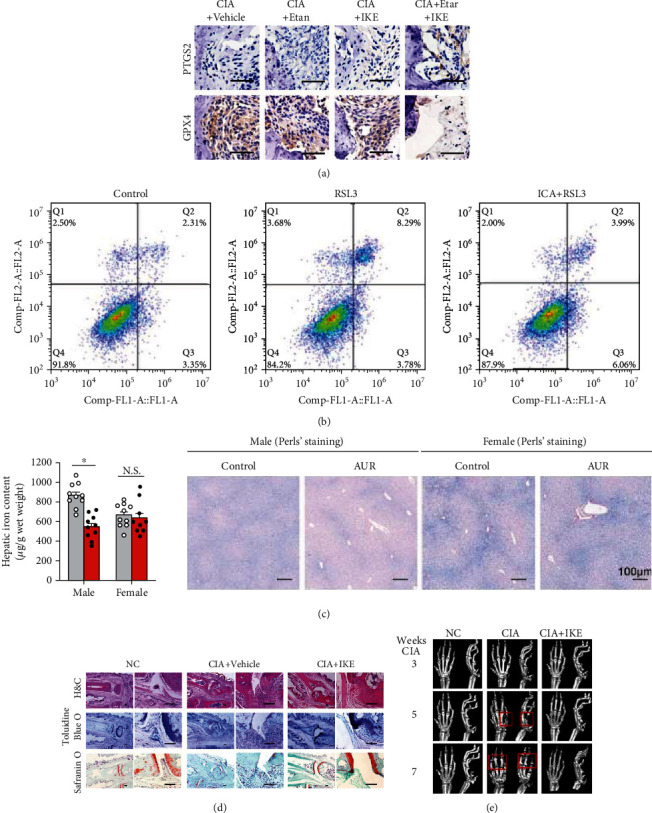
(a) Immunohistochemical staining about PTGS2 and GPX4 expression in the joints of CIA mice (reproduced from ref. [131] with permission from 2022 Nature Publishing Group); (b) cell death in the different study groups by flow cytometry (reproduced from ref. [136] with permission from 2021 Ingenta PLC); (c) Perls' Prussian blue–stained liver sections (reproduced from ref. [138] with permission from 2020 Nature Publishing Group); (d) images of hematoxylin and eosin (H&E), toluidine blue O, and safranin O staining of representative joints in control and CIA mice with or without IKE treatment at day 22 after treatment initiation (reproduced from ref. [131] with permission from 2022 Nature Publishing Group); (e) representative microcomputed tomography (micro-CT) images of control and CIA model mice with or without IKE treatment (reproduced from ref. [131] with permission from 2022 Nature Publishing Group).

Recent studies have shown that ferroptosis plays an important regulatory role in autoimmune and inflammatory diseases [129, 130]. For example, treatment with the Gpx4 inhibitor RSL3 specifically increased cell death in fibroblast activation protein-*α* (FAP*α*+) fibroblasts, but not macrophages, endothelial cells, T cells, or B cells of cell death. In addition, the number of surviving FAP*α*+ fibroblasts in the synovial area was higher, close to that of macrophages, suggesting that macrophages may protect FAP*α*+ fibroblasts from IKE treatment-induced lipid peroxidation and ferroptosis in CIA mice [131]. Of course, how immune cells induce ferroptosis in RA requires further study. Besides, a previous study revealed that in an RA model, metalloproteinases (MMPs) are activated by excessive ROS, thus suppressing the synthesis of cartilage protein and leading to cartilage injury and bone destruction. In short, excessive ROS is closely related to RA [132]. Simultaneously, excessive ROS also have a hand in ferroptosis of synovial cell death. In ferroptosis, ROS are transformed into hydrogen peroxide through the Fenton reaction, which produces hydroxyl (·OH) or alkoxyl (RO^·^) radicals with the help of superoxide dismutase in the presence of reduced Fe^2+^. Afterwards, Fe^3+^ can be converted into Fe^2+^ by the Haber-Weiss reaction [133]. Meanwhile, FSP1 improves lipid peroxidation and blocks iron sagging by combining with CoQ_10_ [134, 135]. It could be calculated that FSP1, which acts parallel to GPX4, is likely to abolish the TNF-*α*/ROS feedback loop and prevent ferroptosis of cell death in RA. Moreover, low-dose imidazole ketone erastin (IKE) together with etanercept, a TNF antagonist, induced ferroptosis in fibroblasts and attenuated arthritis progression in a collagen-induced arthritis (CIA) mouse model (Figures 6(a), 6(d), and 6(e)) [131]. Luo and Zhang and Zu et al. also observed the same phenomenon as ICA, an important role in both rheumatoid arthritis and osteoarthritis and associated with gene expression and cellular functions in the synoviocytes of osteoarthritis, inhibiting ferroptosis through the Xc^−^/GPX4 axis, thus attenuating cell death in the RA model (Figure 6(b)) [136, 137]. On the basis of previous research, Yang *et al.* observed lipid peroxidation and iron metabolism disorders in LPS-induced synovial cells (Figure 6(c)) [138]. At present, many scholars have invested much time and energy in the study of ferroptosis in RA and have developed related drugs, such as curcumin and baicalein, to intervene in RA due to lipid peroxidation and iron metabolism disorders in RA [139, 140]. However, we still know little about the detailed mechanisms. We hope that subsequent research can overcome these bottlenecks.

## 8. Questions and Perspectives

In recent years, our understanding of ferroptosis has gradually deepened in biomedicine, and thousands of articles have been published. On the whole, ferroptosis is considered to be a programmed regulation of cell death, which is strictly regulated at multiple layers and multiple levels [141, 142]. Many pharmacological and genetic operations have been used to regulate changes induced by ferroptosis in multidisciplinary diseases such as cardiovascular diseases, renal injury, and skeletal muscle diseases and attenuate disease mortality and disability rates [43, 138, 143, 144]. However, research on ferroptosis is in an immature stage, and an array of doubts remain unanswered, especially in skeletal diseases. For example, cell death patterns have many similarities in skeletal diseases, such as ferroptosis, autophagy, and apoptosis. What is the association between these cell death patterns? Is it mutual promotion or antagonism? How these different cell death patterns can be integrated into a system still needs further study [6]. According to previous research, iron plays an indispensable role in the development of ferroptosis [1]. With the exception of iron ions, ferroptosis occurs under the regulation of some metal ions in some instances [145, 146]. This makes us doubt the traditional definition of ferroptosis. Is iron vital to promote lipid peroxidation, or can other fungi induce ferroptosis? This view requires further discussion. Subsequent studies have shown that FPN, as an upstream iron metabolism gene, can regulate ferroptosis, but how the downstream pathway is regulated is still not very clear [11, 75–77]. Ferroptosis accompanies inflammation in some diseases such RA and acute kidney injury and modulates the immune system, causing inflammatory damage and inhibiting cell growth [147–150]. Under what circumstances will it promote ferroptosis-induced inflammation? In addition, no clinical trials have been conducted on ferroptosis activators in skeletal diseases. How can we integrate basic research results and thus promote the recovery of skeletal diseases to reduce disability and mortality? As stated above, even if we invest more time and enthusiasm than before in conducting research on ferroptosis, a series of detailed problems about ferroptosis urgently need to be solved.

## 9. Conclusion

In this review, we summarize the mechanism of ferroptosis, such as suppressing GPX4 expression and activating the lipid metabolism pathway; briefly list several inducers and inhibitors; and expound on the manifestations of iron death in skeletal diseases. As will be readily seen from this article, our research on ferroptosis is still superficial at present. It is of great significance to explore the mystery of ferroptosis and its specific role in multiple distinct diseases, especially skeletal diseases, and to develop targeted therapeutic regimens. This will be the general trend of future research.

## Figures and Tables

**Table 1:** Comparison of cell death in ferroptosis, autophagy, and apoptosis.

Comparison of characteristics of cell death in ferroptosis, autophagy, and apoptosis			
Cell death types	Ferroptosis	Autophagy	Apoptosis
Morphological characteristics	Smaller mitochondria, decreased mitochondrial ridge	Autolysosome	Cells became round, chromatin is condensed and fragmented, and cytoplasm shrunk
Other features	Iron ion aggregation, cell membrane rupture	No obvious changes in the nucleus and membrane	Cell shrinks, cytoplasm flows out, and membrane vacuoles
Detection index	ROS, PTGS↑; NADPH↓	LC3-I→LC3II	Caspase↑; intracellular Ca^2+^↑
Positive regulatory factor	Erastin, RSL3, RAS, Sorafenib, p53	ATG family, Beclin1	P53, Bax, Bak, TGF-*β*, radiation, dexamethasone
Negative regulatory factor	GPX4, FSP1, SLC7A11, Nrf2, ferrostatin-1, liproxstatin-1, DFO	mTOR, 3-methyladenine, wortmannin, Spautin1	Bcl-2, Bcd-XL, Z-VAD-FMK, IL-4

**Table 2:** Overview of inducers of ferroptosis.

Target	Inducer	Mechanisms associated with ferroptosis
System Xc^−^	Erastin	Inhibit system Xc^−^ activity
	Erastin2	Inhibition of system Xc^−^ cystine/glutamate transporter
	Imidazole ketone Erastin	Metabolic stabilization inhibitor of system X-
	Glutamate	Inhibit system Xc^−^ activity
GPX4	RSL3	GPX4 bound to selenocysteine sites
	DPI7 (ML162)	Covalently bind GPX4 (same binding site as RSL3)
	DPI10 (ML210)	Indirectly inhibit GPX4 activity or bind to sites different from RSL3
	Altretamine	Inhibit GPX4 activity
GSH	Buthionine sulfoximine	Reduce GSH synthesis
	N-Acetyl-4-benzoquinone imine	Toxic doses deplete glutathione reserves in the liver
	Cisplatin	Binding to GSH inactivates GXP4
	DPl2	Excessive consumption of GSH
	Piperlongumine	Consume GSH and inhibit GPX4 activity
ROS and iron ions	Heme	Increase of intracellular unstable iron
	Withaferin A	Medium dose upregulated HMOX1 expression and increased intracellular unstable iron. High dose inhibited GPX4 activity
	BAY 11-7085	Upregulation of HMOX1 expression and increase of intracellular unstable iron
	FINO_2_	Oxidation of Fe^2+^ promotes ROS accumulation in cells
	Artesunate	Induce ferritin autophagy and release unstable iron
	Dihydroartemisinin	Induce ferritin autophagy and release unstable iron; binding to free iron inhibits ferritin translation
	Siramesine	Decrease the expression of FPN, increased the expression of transferrin, increased the intracellular unstable iron
	BAY 87-2243	Inhibit mitochondrial respiratory chain complex 1 and increase ROS
	iFSP1	Inhibition of FSP1 inhibits ferroptosis unrelated to glutathione activity
ROS	Auranofin	Inhibit thioredoxin reductase activity
	Statins	Inhibits HMG-COA reductase, which catalyzes rate-limiting steps of the MVA pathway
ROS and GSH	QD-394	Induce lipid peroxidation and decrease GSH/GSSH ratio
ROS and SQS	FIN56	Induce GPX4 degradation. Bind and activate SQS to reduce CoQ10

**Table 3:** Overview of inhibitors of ferroptosis.

Target	Inhibitor	Mechanisms associated with ferroptosis
ROS and iron ions	Minocycline	Minocycline reduces iron overload after ICH and iron induced brain injury
	Ferrostain-1	Scavenge ROS, inhibit lipid peroxidation, and reduce unstable iron in cells
	Liproxstatin-1	Scavenge ROS, inhibit lipid peroxidation, and activate the Nrf2 signaling pathway
	Curcumin	Chelate iron, reduce iron accumulation, and activate the Nrf2 signaling pathway
	Alpha tocopherol analogs	Remove ROS and inhibit lipid peroxidation
	Nitrogen oxides	Inhibit Fenton reaction and hydroxyl radical production
GSH and GPX4	Baicalein	Inhibit GSH depletion, GPX4 degradation, and lipid peroxidation and activate the Nrf2 signaling pathway
	Gastrodin	Inhibit glutamate-induced iron death in HT-22 cells
ACSL4	Rezulin	Prevention of ferroptosis and lipid peroxidation in Pfa1 cells induced by RSL3
5-LOx	Zileuton	Protect ACSL4 overexpressed LNCaP and K562 cells from erastin-induced ferroptosis
TFR1 and FTH1	HSPB1	Inhibit ferroptosis induced by erastin
Lipid peroxidation	XJB-5-131	Suppress lipid peroxidation
Iron	Deferoxamine	Deplete iron and prevent iron-dependent lipid peroxidation

## Data Availability

The authors confirm that the data supporting the findings of this study are available within the article.

## Abbreviations

TFR1: Transferrin receptor 1
PUFA: Polyunsaturated fatty acid
LOXs: Lipoxygenase
STEAP3: Six-transmembrane epithelial antigen of prostate 3 metalloreductase
SLC7A11: Solute carrier family 7 member 11
DMT1: Divalent metal transporter 1
SLC3A2: Solute carrier family 3 member 2
BAP1: BRCA1-associated protein 1
ROS: Reactive oxygen species
FSP1: Ferroptosis suppressor protein 1
FPN1: Ferroportin 1
GPX4: Glutathione peroxidase 4
GSH: Glutathione
GSSG: Oxidized glutathione
GSS: Glutathione synthetase
PHKG2: Phosphorylase kinase G2
HSPB1: Heat shock protein beta-1
NCOA4: Nuclear receptor coactivator 4
RSL3: Ras-selective lethal 3
RSL5: Ras-selective lethal 5.
